# Blastic Plasmacytoid Dendritic Cell Neoplasm: State of the Art and Prospects

**DOI:** 10.3390/cancers11050595

**Published:** 2019-04-28

**Authors:** Maria Rosaria Sapienza, Alessandro Pileri, Enrico Derenzini, Federica Melle, Giovanna Motta, Stefano Fiori, Angelica Calleri, Nicola Pimpinelli, Valentina Tabanelli, Stefano Pileri

**Affiliations:** 1 Division of Diagnostic Haematopathology, European Institute of Oncology, IRCCS, Via Ripamonti 435, 20141 Milano, Italy; mariarosaria.sapienza@ieo.it (M.R.S.); federica.melle@ieo.it (F.M.); giovanna.motta@ieo.it (G.M.); stefano.fiori@ieo.it (S.F.); angelica.calleri@ieo.it (A.C.); valentina.tabanelli@ieo.it (V.T.); 2 Unit of Dermatology, Department of Experimental, Diagnostic and Specialty Medicine, University of Bologna, School of Medicine, Via Massarenti 1, 40138 Bologna, Italy; alessandro.pileri2@unibo.it; 3 Division of Haematology, European Institute of Oncology, Via Ripamonti 435, 20141 Milano, Italy; enrico.derenzini@ieo.it; 4 Dermatology Unit, Department of Health and Science, University of Florence, School of Medicine, Viale Michelangiolo 104, 50100 Firenze, Italy; nicola.pimpinelli@unifi.it

**Keywords:** blastic plasmacytoid dendritic cell neoplasm, clinics, morphology, phenotype, gene expression profile, mutational landscape, chemotherapy, targeted therapy

## Abstract

Blastic plasmacytoid dendritic cell neoplasm (BPDCN) is an extremely rare tumour, which usually affects elderly males and presents in the skin with frequent involvement of the bone-marrow, peripheral blood and lymph nodes. It has a dismal prognosis, with most patients dying within one year when treated by conventional chemotherapies. The diagnosis is challenging, since neoplastic cells can resemble lymphoblasts or small immunoblasts, and require the use of a large panel of antibodies, including those against CD4, CD56, CD123, CD303, TCL1, and TCF4. The morphologic and in part phenotypic ambiguity explains the uncertainties as to the histogenesis of the neoplasm that led to the use of various denominations. Recently, a series of molecular studies based on karyotyping, gene expression profiling, and next generation sequencing, have largely unveiled the pathobiology of the tumour and proposed the potentially beneficial use of new drugs. The latter include SL-401, anti-CD123 immunotherapies, venetoclax, BET-inhibitors, and demethylating agents. The epidemiologic, clinical, diagnostic, molecular, and therapeutic features of BPDCN are thoroughly revised in order to contribute to an up-to-date approach to this tumour that has remained an orphan disease for too long.

## 1. Definition

Blastic plasmacytoid dendritic cell neoplasm (BPDCN, ICD-O code 9727/3) is regarded as an orphan tumour due to its rareness and usual clinical aggressiveness with poor response to conventional chemotherapies [1]. It derives from precursors of plasmacytoid dendritic cells (pDCs), also known as professional type I interferon-producing cells or plasmacytoid monocytes. In the Revised WHO Classification of Tumours of Haematopoietic and Lymphoid Tissues, BPDCN is quoted after acute myeloid leukaemia [1]. This reflects the fact that the gene signature of the cell of origin is much closer to myeloid than lymphoid precursors [2].

## 2. BPDCN and pDCs Ontogenesis

Normal pDCs–originally described by Lennert and Remmele [3]-have been variously named through time (e.g., T-associated plasmacells, plasmacytoid T-cells), thus reflecting the uncertainties about their origin (definitely assessed only in the 1990s by Facchetti et al., [4] and their frequent occurrence in the paracortex of reactive lymph nodes. Analogously, the origin of BPDCN remained obscure for many years. In 2008, precursors of plasmacytoid dendritic cells (pDCs) were finally recognized as the normal counterpart of BPDCN [5,6]. Further studies indicated that BPDCN is related to resting pDCs of myeloid origin [2].

At the time being, it is widely accepted that normal pDCs can recognise either myeloid or lymphoid derivation. Both common dendritic cell progenitors (CDPs) and common lymphoid progenitor (CLPs) can differentiate into pDCs, with the same ability to produce type 1 interferons (IFN-I), although only pDCs of myeloid origin can process and present the antigen [7]. CDPs and CLPs derive from bone marrow hematopoietic stem cells (HSCs) through a complex transcriptional network, in which the progressive lineage commitment causes alternative cell fate acquisition. The commitment to pDC lineage is determined by the combinatorial dosage of specific transcription factors driving the transition from myeloid and lymphoid hematopoietic progenitors to differentiated cells [8]. So far, *TCF4*, *BCL11A* and *IRF8* have been regarded as the main transcription factors determining the pDC development. The E-box transcription factor *TCF4* controls the differentiation to the pDC lineage and its maintenance [9]. The B-cell lymphoma/leukemia 11A, *BCL11A*, is essential for pDCs generation and, along with *TCF4*, is used to distinguish the gene expression profile of pDCs from myeloid dendritic cells that, on contrary, are negative for *BCL11A* and highly positive for the B-cell lymphoma 6 protein (*BCL6*) [10]. The IFN regulatory factor 8, *IRF8*, induces in the lymphoid-primed multipotent progenitors and in CDPs the early commitment towards DC lineage and its expression increases during this transition [8,11]. *IRF-8* regulates different hematopoietic lineages and, if mutated, may cause pDC cytopenia and global immunodeficiency [12].

As normal pDCs, BPDCN cells express *BCL11A* and *TCF4* [13], while *IRF8* may be found mutated or mis-spliced [14,15]. The functional consequences of these modifications remain elusive, as does the ability of the tumour to produce IFN-I. BPDCN extensively expresses the interferon-induced GTP-binding protein MxA, used as a surrogate marker of IFN-I on immunohistochemistry [16]. However, after in vitro stimulation not all primary neoplastic cells secrete IFN-I [17] and the BPDCN derived CAL-1 cell line has provided discordant results [18,19,20,21]. Thus, the ability to secrete IFN-1 remains a central issue to clarify the histogenesis of BPDCN and needs systematic investigation in the future.

## 3. Synonyms

The condition has been given several names, such as NK-cell lymphoma, CD4+ NK cell leukaemia or blastic NK leukaemia/lymphoma. All of them are obsolete and reflect the misinterpretation of the histogenesis due to the expression of the CD56 molecule, which is also observed in NK lymphocytes and derived tumours [1]. The term agranular CD4+ CD56+ hematodermic neoplasm/tumour is still in use, although it highlights only some of the diagnostic features and provides no indication as to the histogenesis of the process [1].

## 4. Epidemiology

BPDCN more often affects males (male-to-female ratio = 3.3:1) in the seventh or eighth decade of life, although it can occur at any age, including childhood. Its incidence is 0.000045% [1,22].

## 5. Etiology

There are no data concerning the etiology of BPDCN, except for a certain association with myelodysplastic syndromes (MDS) and MDS/myeloproliferative neoplasms (MPN), with special reference to chronic myelomonocytic leukaemia, a fact that is not surprising taking into consideration the co-occurrence of several gene mutations (see below) [1].

## 6. Clinics

**Figure 1:**
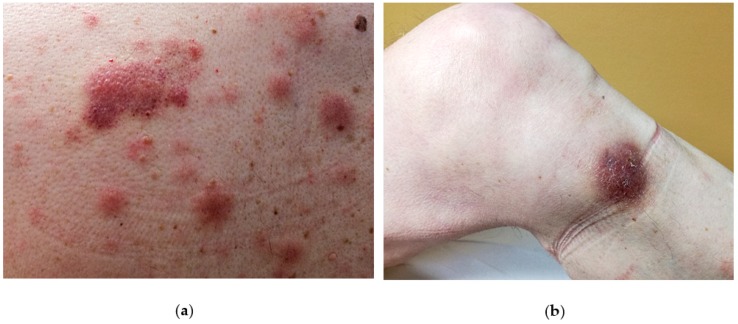
Examples of cutaneous manifestations of BPDCN (**a**) Erythematous-cyanotic plaques on the back of a patient with widespread disease. (**b**) Erythematous-purplish single nodule on the leg of a patient with localized disease.

The disease tends to involve multiple sites [1]. More often, it affects the skin (in 60–100% cases), followed by the bone-marrow and peripheral blood (in 60–90% of cases) and lymph nodes (in 40–50% of cases). In the natural history of the disease, the skin is the first affected site [23,24,25,26,27,28] (90% of patients), where it usually remains confined until a rapid, second step (i.e., leukemic spread and multiorgan involvement) occurs, eventually leading to death. It has been hypothesised that the skin may act as a sanctuary organ limiting the disease spread at the beginning [29]. However, a few cases lacking the initial cutaneous involvement have been reported in the literature [30,31,32,33,34]. BPDCN cutaneous tropism has been related to the expression of skin-migration molecules such as CLA and CD56 by the neoplastic elements. Another possible explanation may be the local availability of chemokines binding cognate receptor expressed by the neoplastic cells such as CXCR3, CXCR4, CXCR6, CXCR7. At skin level, the disease can present as isolated or disseminated bruise-like lesions [27,35]. The lesions are usually described as erythematous to purplish papules, plaques or tumours with a heterogeneous size (from few millimetres to several centimetres) with no preferred anatomic area (Figure 1) [27,35]. On clinical grounds, an important distinction should be made between the presence of isolated and eruptive lesions [29]. The former have a better clinical outcome, while the latter should be regarded as a marker of an aggressive disease (progression free survival of 23 vs. 9 months, respectively) [29]. Theoretically, the different behaviour may be due to a high tumour burden in the eruptive presentation. Cases featuring mucosal involvement, especially in the oral cavity have rarely been observed [28].

## 7. Microscopic Findings

**Figure 2:**
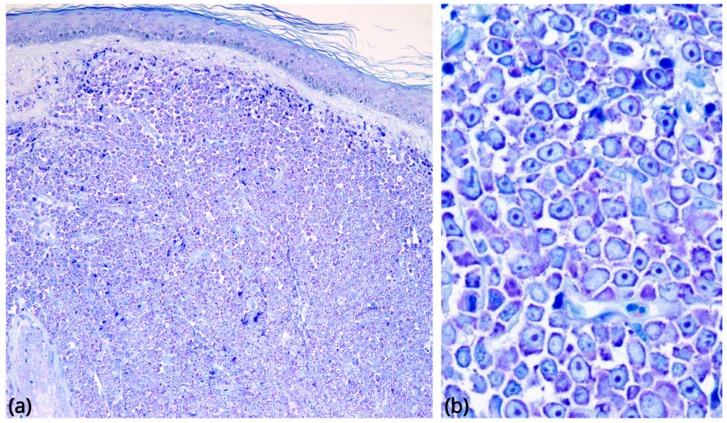
Morphological findings in BPDCN. (**a**) Skin involvement, low-power Giemsa stain (original magnification 40×): a diffuse, monomorphous infiltrate massively involves the dermis, without epidermotropism. (**b**) The neoplastic cells are medium-sized blasts with fine chromatin and scanty cytoplasm, agranular on Giemsa staining (original magnification 600×).

In its most common form, BPDCN is characterized by a diffuse, monomorphous infiltrate of medium-sized blasts reminiscent of either lymphoblasts or myeloblasts [1]. Nuclei have a slightly irregular profile, fine chromatin, and from one to several small nucleoli. The cytoplasm rim is usually narrow and turns greyish-blue and agranular on Giemsa staining. Mitoses are variable in number, and the Ki-67 rate ranges from 20 to 80% (Figure 2). Recently, a morphologic variant provided with immunoblastic-like appearance has been reported in association with *MYC* rearrangement [36]. Angioinvasion and coagulative necrosis are absent [27,37]. In the skin, the dermis is usually massively infiltrated, with extension to the subcutaneous fat. The epidermis and adnexa are generally spared [27]. In lymph nodes, there is diffuse involvement of the interfollicular areas and medulla, B-cell follicles being more often spared. Bone marrow biopsy shows either a subtle interstitial infiltrate (detectable only by immunohistochemistry) or-more often-massive replacement; residual haematopoiesis may display dysplastic changes, especially in megakaryocytes [38]. On peripheral blood and bone-marrow smears, tumour cells may show cytoplasmic microvacuoles localized along the cell membrane and pseudopodia [39].

## 8. Cytochemistry

Neoplastic cells are negative for myeloperoxidase, α-naphthylbutyrate esterase, ND naphthol AS-D chloroacetate esterase.

## 9. Immunophenotype

**Figure 3:**
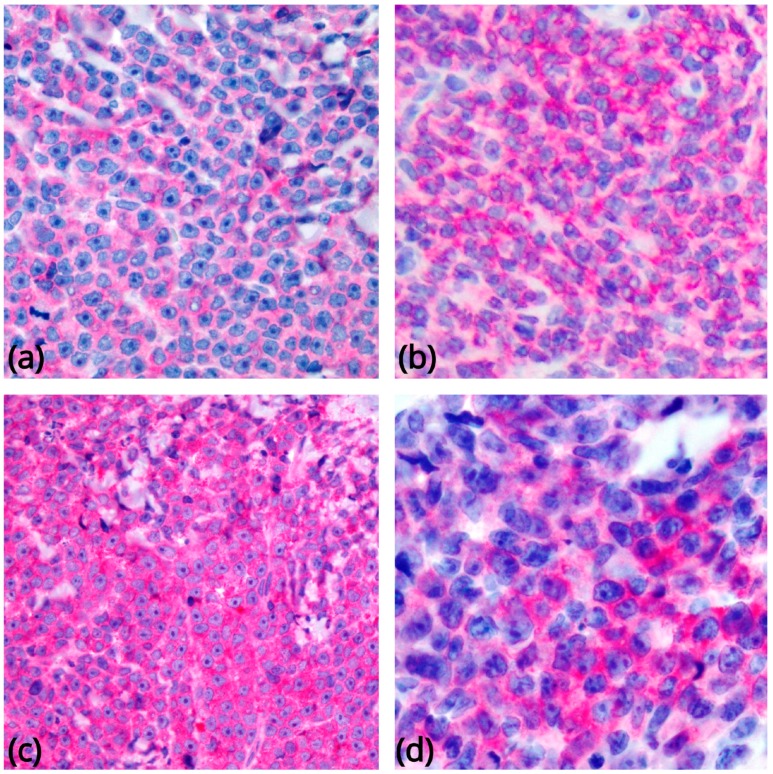
Most common phenotypic findings in BPDCN. Tumor cells show immunoreactivity for CD4 (**a**), CD56 (**b**), CD123 (**c**), and CD303 (**d**) (original magnification 400×).

BPDCN cells express CD4, CD43, CD45RA, and CD56, along with the pDC associated antigens CD123 (IL3 α chain receptor), CD303, TCL1A, CD2AP, and SPIB, and the type I interferon– dependent molecule MX1 [16,24,37,38,40,41,42,43,44,45] (Figure 3). Recently, the TCF4 (E2-2) transcription factor, essential for PDC development, has been reported as a reliable diagnostic marker for BPDCN [13]. About 8% of cases display CD4 or CD56 negativity, a fact that does not rule out the diagnosis in case other pDC-associated antigens are detected [37,41,46]. CD68 (an antigen typically expressed on normal pDCs) is detected in 50–80% of cases, in the form of small cytoplasmic dots [24,38]. CD7 and CD33 are relatively commonly expressed; some cases turn positive for CD2, CD5, CD36, CD38, and CD79a, while CD3, CD13, CD16, CD19, CD20, LAT, lysozyme, and MPO are regularly negative. Granzyme B, which is found in normal pDCs, has also been demonstrated by FACS and mRNA analyses [17,47], but it is typically negative on tissue sections, as are other cytotoxic molecules such as perforin and TIA1. Besides CD56, BPDCN may also express other antigens negative in normal pDCs, including BCL6, IRF4, and BCL2 [27] (the latter potentially acting against tumour cell apoptosis) [2]. S100 protein is expressed in 25–30% of cases [28], and even more frequently in the pediatric ones [48,49]. TdT is positive in about one third of cases, with expression in 10–80% of the cells [37,43,50,51,52]. Occasionally, BPDCN carries KIT (CD117). CD34 is negative on sections [17,27,38,52,53,54] but has been found by FACS analysis in 17% of cases [55]. The search for EBV is always negative. Among the antigens generally expressed by BPDCN blasts, CD123 could serve as a therapeutic target of engineered monoclonal antibodies [56,57,58,59]. As other haematological neoplasms can share morphological and immunophenotypic features with BPDCN (especially AML with monocytic differentiation, which can carry CD4, CD56, and CD123 [41,60,61], extensive immunohistochemical and/or genetic analysis is required before a definitive diagnosis of BPDCN can be made. BPDCNs must also be distinguished from mature pDC proliferations (MPDCPs) associated with other myeloid neoplasms, which are predominantly found in lymph nodes, skin, and bone marrow. MPDCPs consist of nodules or irregular aggregates composed of cells morphologically and phenotypically similar to normal PDCs, frequently undergoing apoptosis. Occasionally, they can reveal aberrant single or multiple antigen expression [43,61,62,63,64]. CD56 is negative in most instances or shows only focal and weak reactivity [61,65]. MPDCPs are characterized by a low Ki-67 proliferation index (<10%) and lack TdT. Their neoplastic nature and relatedness with the associated myeloid neoplasm have been evidenced by the demonstration of identical clonal chromosomal abnormalities in the two cellular components [64,66,67].

## 10. Genetics

### 10.1. Karyotyping

BPDCN patients are affected by frequent chromosomal alterations: up to 75% of them present a complex karyotype (≥3 abnormalities). Conventional cytogenetics studies reported a prevalence of genomic losses on gains and recognized six recurrent deletions of the regions 5q21 or 5q34 (72%), 12p13 (64%), 13q13-21 (64%), 6q23-qter (50%), 15q (43%) and of the entire chromosome 9 (28%) [52,68]. Although recurrent, none of these alterations turned out to be BPDCN-specific, also being observed in other hematological malignancies.

By FISH analysis, *MYC* translocations were reported in the 39% of BPDCN patients, in association with the above mentioned immunoblast-like morphology [36]. T(6;8)(p21;q24) corresponded to the commonest type of *MYC* rearrangement: it defined a subgroup of patients with a more aggressive behavior [69]. Of clinical relevance, MYC positivity was found to confer good response to the acute lymphoblastic leukemia (ALL)–based chemotherapy in a limited number of patients [36,70]. Furthermore, FISH analysis documented in a few cases the translocation of the *MLL1* gene, also recorded in 18% of ALLs [71,72,73,74], along with frequent rearrangements of the ETS variant gene 6 (*ETV6*), a transcription factor disrupted in other hematological malignancies [75,76].

Akin to cytogenetics and FISH, a-CGH evidenced frequent deletions on the chromosomes 4, 9 and 13. Lucioni et al. analyzed by a-CGH 21 BPDCNs and found that the most affected chromosomes were Chr 9 (71%), 13 (61%), 12 (57%), 5 (19%), 7 (19%), 14 (19%), and 15 (14%). The deletions outnumbered the amplifications and resulted in the loss of *CDKN1B*, *CDKN2A*, *CDKN2B*, *RB1*, *LATS2*, and *IKZF1* [77]. While aberrations of *IKZF1,* which is involved in the regulation of dendritic cell hematopoiesis, may cause pDCs deficiency [78], their impact on BPDCN (see below) remains indeterminate yet. The biallelic deletion of *CDKN2A* predicted a worse survival outcome [77]. Wiesner et al. performed a-CGH and immunostaining analysis of 14 BPDCN skin samples and confirmed recurrent deletions along chromosomes 9, 12, 13, and 15, combined with the negative or weak expression of multiple cell-cycle and tumor suppressor genes (e.g., *CDKN1B*, *CDKN2A*, *RB1* and *TP53*) [79], possibly responsible for the uncontrolled proliferation and aggressiveness of BPDCN tumor cells [79,80].

### 10.2. Gene Expression Profiling by Array

The first study of BPDCN gene expression profiling (GEP) was conducted in 2007 by Dijkman et al. Since BPDCN skin lesions could easily be confused with cutaneous myelomonocytic leukemia (c-AML), Dijkman et al. performed a-CGH and GEP by array of 5 BPDCN skin biopsies and 6 c-AML cases. According to their study, BPDCN displayed: (1) a transcriptome profile and a molecular karyotype indeed distinct from c-AMLs; (2) recurrent deletions of 4q34, 9, and 13q12-q31 chromosomal regions; (3) lower expression of *RB1* and *LATS2* tumor suppressor genes; (4) higher expression of various pDC-related genes, such as the *TLRs*, *TLR9* and *TLR10* [60]. In 2014, Sapienza et al. compared for the first time the gene signature of 27 BPDCN primary samples with that of normal pDCs and found that the tumor transcriptome was more similar to resting pDCs rather than activated ones, confirming at molecular level the origin of BPDCN from a pDC precursor [2]. Tumor samples displayed 142 differentially expressed genes, mostly upregulated (89%), including those encoding for CyclinD1 and the anti-apoptotic protein BCL2. Bioinformatic analysis of GEP data revealed the aberrant activation of the NF-kB pathway, a finding suggesting possible response of BPDCN samples/cell lines to the proteasome inhibitor Bortezomib [2]. In vitro and in vivo experiments demonstrated that Bortezomib successfully shuts-down the NF-kB pathway and significantly induces BPDCN cell apoptosis, providing a potential new therapeutic option for BPDCN patients [2,81].

Ceroi et al. performed transcriptional profiling of 12 BPDCN cases by array and focused on a specific signature of downregulated genes involved in cholesterol homeostasis and responsible for its accumulation within the tumor cells. These sets of downregulated genes, if activated, stimulated the cholesterol efflux from neoplastic cells, inhibited the NF-kB pathway and arrested the BPDCN tumor cell survival [82].

### 10.3. Sequencing Studies

The chromosomal lesions of BPDCNs fully reflect their myeloid origin and the same could be expected at the DNA mutational level. Starting from the premise that the mutations of the epigenetic regulator gene *TET2* are diffused in the myeloid lineage [83], Jardin et al. decided to explore the mutational status of this gene in 13 BPDCNs. *TET2* was mutated in more than half of patients and was mostly affected by deleterious mutations (frameshift or nonsense). At diagnosis, *TET2* mutations (54%) were recurrently flanked by *TP53* mutations (38%) leading to hypothesize a synergistic effect between the two genes [84]. Alayed et al. confirmed the high mutational frequency of *TET2* in BPDCN [46]. Ladikou et al. conducted the first targeted-sequencing on the BPDCN circulating free DNA of BPDCN cases, by identifying novel mutations of *TET2* and *RHOA* [85]. Besides *TET2*, thanks to the targeted sequencing approach, many other myeloid-associated genes have been investigated in BPDCN. Taylor et al. presented to the ASH Meeting the first study of targeted sequencing of 219 myeloid-related genes in seven BPDCN samples. The most frequently mutated gene was the splicing factor *ZRSR2* (57%) *ex aequo* with *TET2* (57%), followed by *ASXL1*, *TP53* and *IDH2*, *KRAS*, *ABL1*, *ARID1A*, *GNA13*, *U2AF1*, *SRSF2*, and the transcription factor *IRF8* associated with dendritic cell deficiency [14]. Later, Stenzinger et al. sequenced 50 common myeloid genes in 33 cases of BPDCN and, in order of prevalence, detected somatic mutations on *NRAS*, *ATM*, *KRAS*, *MET*, *IDH2*, *KIT*, *RB1*, *APC*, *TP53*, *RET*, *VHL*, *BRAF*, and *MLH1* genes, and deletions of *CDKN2A*, *RB1*, *PTEN*, and *TP53* genes, already found by a-CGH [86]. Menezes et al. analyzed three patients by whole exome sequencing (WES) and used the WES results to design a targeted-sequencing panel of selected genes to examine 38 BPDCN samples. The most affected genes were *TET2* (36%), *ASXL1* (32%), *NRAS*, *NPM1*, and *IKZF* family 1/2/3 (20%). Overall, 50% and 20% of patients with mutations in genes encoding for epigenetic factors or belonging to the *IKAROS* family respectively experienced a significantly reduced overall survival [87].

More recently, integrated “omics” approaches have been applied aiming to better understand the tumor biology. Montero et al. analyzed, by RNA-sequencing, 12 BPDCN samples and four pDCs from healthy donors by confirming *BCL2* overexpression in tumors. Furthermore, by the BH3-proling of two BPDCN cell lines (CAL-1 and GEN2.2), six primary patient samples, and six patient-derived xenografts, the same authors demonstrated the BCL2 dependence of BPDCN elements as well as their sensitivity to the BCL2 inhibitor venetoclax. In the light of this finding, two patients were then treated with venetoclax and experienced significant disease responses [88,89].

Ceribelli et al. first performed an RNA interference screening study of the CAL-1 BPDCN cell line and recognized the transcription factor *TCF4* as a master regulator of the BPDCN oncogenic program: its downregulation provoked the loss of the BPDCN-specific gene expression signature along with tumor cell death. Already described as relevant in normal pDC development, the *TCF4* gene product was positively detected by immunohistochemistry in all the 28 BPDCN samples examined and proposed as a new reliable diagnostic marker (see above) and potential therapeutic target for bromodomain and extra-terminal domain inhibitors (BETis) [13].

Emadali et al. further substantiated the use of BETis in BPDCN. They examined 47 tumor samples and the CAL-1 cell line by various techniques (e.g., cytogenetics, a-CGH, FISH, targeted sequencing) and found that the loss of the glucocorticoid receptor gene, *NR3C1*, defined a high-risk group of patients. *NR3C1* is often juxtaposed with *lncRNA3q*, a novel nuclear noncoding RNA involved in the regulation of leukemia stem cell programs and G1/S transition and aberrantly overexpressed in BPDCN malignant cells. BETis successfully turned-off the expression of *lncRNA3q* and inhibited tumor cells growth [90].

Suzuki et al., used RNA sequencing technology to discover novel fusion genes in 14 BPDCNs corresponding to five children and nine adults; recurrent *MYB* gene rearrangement were identified in all the children (100%) and in four out of the nine adults (44%) [91].

Sapienza et al. analyzed BPDCNs by WES, RNA and Chromatin Immunoprecipitation (ChIP) sequencing approaches. Several epigenetic factor genes were found mutated (e.g., *ASXL1*, *TET2*, *SUZ12*, *ARID1A*, *PHF2*, *CHD8)* and the functional enrichment analysis of the mutational data showed that of all the biological programs explored, the epigenetic was the most affected. At transcriptomic level, the patients displayed the significant enrichment of gene signatures related to epigenetic pathways, predicting response to hypomethylating agents. Accordingly, the use of 5’-azacytidine in combination with decitabine significantly inhibited disease progression and extended survival in a preclinical mouse model [22].

## 11. Therapy of Blastic Plasmacytoid Dendritic Neoplasm

BPDCN is characterized by an inherent resistance to standard chemotherapies. Treatment responses are mostly transient, the overall outcome being general very poor in general [32,54,92]. Given the rarity of the disease, the available data on BPDCN therapy mainly derive from retrospective studies.

In general, intensive induction regimens (e.g., hyperCVAD) are considered more effective compared to standard therapies (e.g., CHOP-like) [39,92,93]. In general, ALL-like treatments seem to be more effective in term of response rates than AML-like induction therapies [32,54,92]. The inclusion of l-asparaginase in ALL-like regimens could be a significant determinant of efficacy in this setting, as l-asparaginase has shown clinical activity in BPDCN in combination with single agent methotrexate [94,95].

Regarding the role of hematopoietic stem cell transplant in BPDCN therapy, there are several reports suggesting better results in terms of enduring remissions and relapse rates with allogeneic-stem cell transplantation (allo-SCT) compared to auto-SCT. These studies demonstrated durable complete remissions with allo-SCT, with OS rates ranging from 40% at 10 years, to 58% at 3 years depending on the follow-up period [96,97]. In general, allo-SCT consolidation seems to yield the best results when performed in first complete remission (CR) [97,98,99], with OS rates reaching 74–82% at 3–4 years [97,99]. Reduced intensity conditioning seems to be equivalent to myeloablative regimens in terms of relapse rates [97]. However, these data should be interpreted with due caution given the possible biases arising from the retrospective nature of these studies (e.g., patient selection bias, absence of intention to treat analyses, small sample size).

Eligible patients should be considered for allo-SCT consolidation in first CR whenever feasible. It should be noted, however, that these patients represent the minority of BPDCN patients, as the disease normally affects elderly patients, with a median age of 68 years [32].

For elderly patients, lower intensity treatments can be explored. Lower intensity chemotherapy regimens demonstrated some efficacy in BPDCN, such as single agent pralatrexate, bendamustine, or gemcitabine/docetaxel combinations [100,101,102,103]. However, despite the promising results, these studies were performed on a small number of patients, and which ought to be validated in larger future studies.

A recent study by our group strongly supports the use of hypomethylating agents, demonstrating a significant enrichment in epigenetic modifiers mutations in the setting of BPDCN [22]. In line with our preclinical findings, two clinical reports have demonstrated activity of 5-azacitidine in BPDCN, although the responses were generally transient once again [104,105]. Combinatory approaches based on hypomethylating agents should be explored in the near future.

### Novel Agents

Given the unsatisfactory results of low-intensity treatments, and the toxicity of intensive therapies and allo-SCT consolidation, there is strong rationale for the use of novel targeted agents for the treatment of BPDCN.

SL-401 is a novel recombinant protein including components of diphtheria toxin fused to interleukin-3. As mentioned in previous sections, CD123 is expressed on the surface of BPDCN cells. In a phase I study of SL-401 in BPDCN the overall response rate was 77% (with 55% CR) in the evaluable patient population (seven out of eleven patients were able to complete the planned treatment) [58]. A phase 2 study reported at the 2017 ASH meeting showed promising results with a 79% CR rate in first line and 31% CR rate in relapsed/refractory patients [106].

Phase I trials are ongoing with other immunotherapies targeting CD123, such as bispecific antibodies, immunoconjugates, and chimeric antigen receptor (CAR)-T-cells [107]. In fact, recent data show promising activity of anti-CD123 CAR T-cells in acute myeloid leukemia and preliminary experiences support the future implementation of anti-CD123 CAR-T cell therapy in the BPDCN setting [108]. The BCL-2 inhibitor venetoclax has shown high single agent activity in myeloid malignancies [109,110] and is currently under evaluation in combination with induction chemotherapy and hypomethylating agents.

Several recently published reports have described the activity of venetoclax in the setting of BPDCN [89,110,111,112]. Venetoclax given as single agent or in combination with hypomethylating agents was able to induce meaningful clinical responses in relapsed/refractory patients. Further therapeutic options may be represented by bromodomain and extra-terminal domain inhibitors (BETis), which has been tested in preclinical studies [13,90].

## 12. Conclusions and Perspectives

Although the criteria for the diagnosis of BPDCN are well-defined [1], the knowledge of the pathobiology of the tumour is still based on a limited number of contributions, which reflect its exceptional occurrence. The epigenetic regulation, activation of the NF-kB pathway, and resistance to apoptosis seem to represent the main biological players, which should be taken into consideration in designing innovative therapeutic strategies. BPDCN is in fact characterized by intrinsic resistance to standard chemotherapies. In young patients, intensive ALL-like induction regimens followed by allo-SCT consolidation is considered the most effective treatment strategy, leading to durable responses in a fraction of cases. Elderly patients (who represent the majority of BPDCN patients) remain an unmet medical need. Recently, hypomethylating agents, anti CD123 directed immunotherapies and the BCL-2 inhibitor venetoclax showed promising single-agent clinical activity. These observations, together with emerging preclinical data provide the rationale for the prompt clinical testing of combinatory approaches with curative intent.
